# Derived Patterns in Binocular Rivalry Networks

**DOI:** 10.1186/2190-8567-3-6

**Published:** 2013-05-08

**Authors:** Casey O Diekman, Martin Golubitsky, Yunjiao Wang

**Affiliations:** 1 Mathematical Biosciences Institute, The Ohio State University, Columbus, OH, 43210, USA; 2 Department of Mathematics, Texas Southern University, Houston, TX, 77004, USA

**Keywords:** Binocular rivalry, Interocular grouping, Coupled systems, Symmetry, Hopf bifurcation

## Abstract

Binocular rivalry is the alternation in visual perception that can occur when the two eyes are presented with different images. Wilson proposed a class of neuronal network models that generalize rivalry to multiple competing patterns. The networks are assumed to have learned several patterns, and rivalry is identified with time periodic states that have periods of dominance of different patterns. Here, we show that these networks can also support patterns that were not learned, which we call *derived*. This is important because there is evidence for perception of derived patterns in the binocular rivalry experiments of Kovács, Papathomas, Yang, and Fehér. We construct modified Wilson networks for these experiments and use symmetry breaking to make predictions regarding states that a subject might perceive. Specifically, we modify the networks to include lateral coupling, which is inspired by the known structure of the primary visual cortex. The modified network models make expected the surprising outcomes observed in these experiments.

## 1 Introduction

Wilson [1] argues that generalizations of binocular rivalry can provide insight into conscious brain processes and proposes a neural network model for higher level decision making. Here, we demonstrate that the Wilson network model is also useful for understanding the phenomenon of binocular rivalry itself by analyzing several rivalry experiments discussed in Kovács, Papathomas, Yang, and Fehér [2]. Mathematical analysis of these network structures (based on the theory of coupled cell systems and symmetry in Golubitsky and Stewart [3-5]) leads to predictions that are directly testable via standard psychophysics experiments. 

**Fig. 1:**
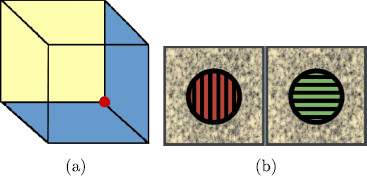
**a** Necker cube illusion [7] and **b** rivalry [6]

We begin by making a distinction between two types of perceptual alternations (Blake and Logothetis [6]): *illusions* due to insufficient information and *rivalry* due to inconsistent information. One of the standard examples of illusion is given by the *Necker Cube* shown in Fig. 1(a). There are two percepts that are commonly *perceived* when viewing the Necker cube picture: one with the yellow face at the back and one with it on top. There is not enough information in the picture to fix the percept and this ambiguity leads to the two percepts alternating randomly. 

In rivalry, the two eyes of the subject are presented with two different images such as the ones in Fig. 1(b) [6]. Typically, the subject reports perceiving the two images alternating in periods of dominance. There are two main types of mathematical models for rivalry (Laing et al. [8]). In the first type, rivalry is treated as a time periodic state (perhaps with added noise), and in the second the oscillation is obtained by noise driven jumping between stable equilibria in a bistable system (Moreno-Bote et al. [9]). 

**Fig. 2:**
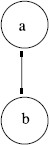
Two-node architecture modeling two units

The simplest deterministic version of the first type, studied by many authors including [1,10-18], assumes that there are two units *a* and *b* corresponding to the two percepts with a system of differential equations of the form

(1) $$\begin{array}{rl} {\dot{X}}_{a} & =F(X_{a},X_{b})\\ {\dot{X}}_{b} & =F(X_{b},X_{a}) \end{array}$$

where the vector $X_{a}$ consists of the state variables of unit *a* and the vector $X_{b}$ consists of the state variables of unit *b*. The equations in (1) are those associated with the two-node network in Fig. 2. It is further assumed that one of the variables $x_{*}^{E}$ is an *activity* variable and that $x_{a}^{E}>x_{b}^{E}$ implies that percept *a* is dominant. Similarly, percept *b* is dominant if $x_{b}^{E}>x_{a}^{E}$. In these models equilibria where $X_{a}\neq X_{b}$ are *winner-take-all* states that correspond to one percept being dominant. 

States where $X_{a}=X_{b}$ are called *fusion* states. Fusion states are typically interpreted as states where a subject perceives both of the images superimposed [18-20]. One might wonder why fusion states would be of interest in mathematical models, since it seems unlikely that the two values $X_{a}$ and $X_{b}$ would be equal. However, because of the symmetry in model equations such as (1), the subspace $X_{a}=X_{b}$ is flow-invariant, and fusion equilibria are structurally stable.

Periodic solutions representing rivalry are most easily found in model equations (1) by using symmetry-breaking Hopf bifurcation from a fusion state. Note that the symmetry in (1) is given by permuting the two units. In such systems, there are two types of Hopf bifurcation: symmetry-preserving and symmetry-breaking. The two types are distinguished by which subspace

$$V^{+}=\left\{(X_{a},X_{b}):X_{b}=X_{a})\right\}\;\text{or}\;V^{-}=\left\{(X_{a},X_{b}):X_{b}=-X_{a})\right\}$$

contains the critical eigenvectors at Hopf bifurcation. Symmetry implies that generically the critical eigenvectors are either in one subspace or the other [5]. Symmetry-preserving Hopf bifurcations (with critical eigenvectors in $V^{+}$) lead to periodic solutions satisfying $X_{b}(t)=X_{a}(t)$, that is, to oscillation of fusion states. These states are perhaps uninteresting from the point of view of rivalry. Symmetry-breaking Hopf bifurcations (with critical eigenvectors in $V^{-}$) lead to periodic solutions satisfying $X_{b}(t)=X_{a}(t+\frac{T}{2})$, where *T* is the period. Such solutions lead to periodic alternation between percepts *a* and *b*; that is, to rivalrous solutions.

**Fig. 3:**
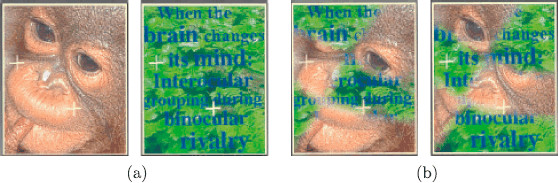
From Kovács et al. [2] ©(1996) National Academy of Sciences, USA. **a** Learned images in *monkey*-*text* rivalry experiment. **b** Learned images in scrambled *monkey*-*text* experiment

Kovács et al. [2] published an influential paper demonstrating that subjects can perceive alternations between coherent images even when the components of those images are scrambled and distributed between the two eyes (Lee and Blake [21]). The unscrambling of component pieces to obtain a coherent percept, termed *interocular grouping*, had been documented previously (Diaz-Coneja [22] and Alais et al. [23]), and has since been reproduced using a variety of rivalry stimuli (Papathomas et al. [24]). Of the four rivalry experiments described in [2], only the first can be understood by the simple two-node network in Fig. 2. We will show that the other three experiments can be modeled using a variant of Wilson networks for generalized rivalry. In their first experiment, subjects are presented the *monkey* and *text* images in Fig. 3(a)) and they report rivalry between the two images. In their second experiment, subjects are presented the scrambled images combining parts of the monkey’s face and parts of the written text (see Fig. 3(b)). The subjects report that, in addition to the expected rivalry between the original scrambled images, for part of the time they perceive alternations between unscrambled images of *monkey* only and *text* only such as those in Fig. 3(a). We show that the surprising outcome of this experiment is not surprising when formulated as a simple Wilson network. 

**Fig. 4:**
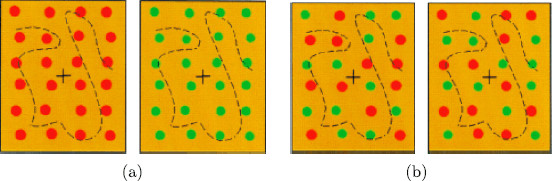
From Kovács et al. [2] ©(1996) National Academy of Sciences, USA. **a** Learned images in conventional *colored dot* experiment. **b** Learned images in scrambled *colored dot* experiment

Kovács et al. [2] also discuss two *colored dot* rivalry experiments that are analogous to the conventional and scrambled *monkey-text* experiments. In the conventional *colored dot* experiment, the subjects were shown the single-color images in Fig. 4(a). Besides reporting rivalry between the two single-color figures, the subjects unexpectedly report images with dots of scrambled colors, such as those in Fig. 4(b). The corresponding result for the conventional *monkey*-*text* experiment seems highly unlikely. 

In the scrambled *colored dot* experiment, [2] presented the subjects with the images in Fig. 4(b). Given the results of the scrambled *monkey*-*text* experiment, it is not surprising that subjects reported rivalry between these two scrambled color images and also rivalry between single color images such as shown in Fig. 4(a). However, the analogy of the scrambled *colored dot* experiment with the scrambled *monkey*-*text* experiment is not quite so straightforward, since, as we will see in Sect. 2, the proposed Wilson networks for the two experiments have different symmetries.

Tong, Meng, and Blake [25] give a simplified description of the *colored dot* experiments of [2] by using a square array of four dots rather than a rectangular array of 24 dots. Our analysis is based on the simplified 2×2 versions of these experiments, but extends to the 6×4 case.

The purpose of this paper is to show that all of the surprising observations made in the four rivalry experiments reported by [2] can be understood by analyzing associated Wilson network models for these experiments. We wish to make the following points. 

(1) The simplest Wilson model for the conventional *monkey*-*text* experiment is the standard two-node rivalry model in Fig. 2 and leads only to rivalry between the whole *monkey* and the whole *text* images.

(2) The simplest Wilson model for the scrambled *monkey*-*text* experiment leads naturally to rivalry solutions between the scrambled images and also between the reconstructed images.

(3) The modified Wilson model for the scrambled *colored dot* experiment also leads naturally to rivalry solutions between both the scrambled and the reconstructed images.

(4) The Wilson model for the conventional *colored dot* experiment leads naturally to rivalry between scrambled images as well as to between the conventional images. This is in contrast to the conventional *monkey*-*text* experiment.

We will see that our analysis also leads to possible additional rivalry states in the *colored dot* experiments and these states may be thought of as predictions made by our approach.

The remainder of the paper is organized as follows. We describe Wilson networks in Sect. 2. Our discussion differs from [1] in two important ways. First, we observe that patterns exist in rivalrous solutions for the Wilson networks that are not learned patterns. We call these additional patterns *derived*; the derived patterns are the ones that correspond to the unexpected results in the Kovács et al. experiments. Second, we introduce an additional type of coupling, *lateral coupling*, based on models of hypercolumns in the primary visual cortex literature (Bressloff et al. [26]). 

Deciding on the exact form of a Wilson network model for a given experiment is not at this stage algorithmic. Moreover, there are many choices for the exact form of the network equations once the network is fixed. If we take the strict form of the Wilson models (where all nodes, all excitatory couplings, and all inhibitory couplings are identical) and we assume that the associated differential equations are highly idealized rate models (as Wilson does), then the derived patterns in the *monkey*-*text* experiment are always unstable. However, stability is a model-dependent property of solutions and simple changes to the network or to the model equations can lead to stable derived patterns.

There are many ways to modify Wilson networks to address the stability issue and we have chosen one here, namely, we have added lateral coupling to the network. Lateral coupling will also enable us to distinguish the Wilson network models for the two *colored dot* experiments by a change in network symmetry. The most important message in this paper is the observation that Wilson networks have derived patterns that can be classified using methods from the theory of symmetry-breaking Hopf bifurcations and that these derived patterns appear to correspond to the surprising perceived states found in psychophysics experiments. More discussion is needed to arrive at an algorithmic description of which (modified) Wilson network to use when modeling a given experiment.

Section 3 gives a brief description of equivariant Hopf bifurcation (see Golubitsky et al. [5]) and shows how to find periodic solutions in Wilson networks modeling the four rivalry experiments that correspond to the rivalries reported in these experiments. Our Hopf bifurcation analysis of the *colored dot* experiments is based on the four-dot version in [25] and on Hopf bifurcation in the presence of $S_{4}$ symmetry (analyzed in Stewart [27]) and of $D_{4}$ symmetry (as in Golubitsky et al. [5]). Note that $S_{4}$ is the group of permutations on four letters and $D_{4}$ is the symmetry group of a square.

Section 4 summarizes the calculations needed to compute stability for rivalrous solutions between both learned and derived patterns in the scrambled *monkey*-*text* networks. In this section, we use standard rate models to compute stability and to illustrate the effect of having lateral coupling.

We end this Introduction by emphasizing that our approach is mainly a *model independent* one advocated in Golubitsky and Stewart [4]. We use network structure and symmetry to create a menu of possible rivalrous solutions, rather than explicitly finding these solutions in a given differential equations model, such as is typically done in the literature [1,10,11,28]. This menu is model independent. Stability, on the other hand, is *model dependent*. Our discussion of stability in Sect. 4 does rely on the choice of specific model equations; here we use the rate models introduced by others.

## 2 Networks

Wilson networks [1] are assumed to have learned several patterns, and rivalry is identified with time-periodic states that have periods of dominance of different patterns. Here, we show that these networks can also support derived patterns in addition to learned patterns. 

**Fig. 5:**
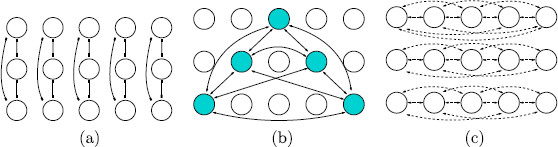
Architecture for a Wilson network. **a** Inhibitory connections between nodes in an attribute column. **b** Excitatory connections in a learned pattern. **c** Excitatory lateral connections

A pattern is defined by the choice of levels of a set of attributes. Specifically, Wilson networks consist of a rectangular set of nodes, arranged in columns, and two types of coupling. The columns represent attributes of an object and the rows represent possible levels of each attribute. There are reciprocal inhibitory connections between all nodes in each column. See Fig. 5(a). In the Wilson network a *pattern* is a choice of a single level in each column. If the network has *learned* a particular pattern, then there are reciprocal excitatory connections between all nodes in the pattern. See Fig. 5(b). A Wilson network can learn many patterns. When it does, there are reciprocal excitatory connections between nodes in each pattern. In our discussion of rivalry, we assume that the images shown to each eye are the two learned patterns. 

Before discussing networks for the rivalry experiments in [2], we consider a variant of Wilson networks that introduces a third type of coupling. This coupling is inspired by the hypercolumn structure of the primary visual cortex (V1). Neurons in V1 are known to be sensitive to orientations of line segments located in small regions of the visual field. Moreover, V1 consists of hypercolumns, which are small regions of V1 that correspond to specific regions of the visual field. Optical imaging of macaque V1 suggests that in each hypercolumn there are neurons that are sensitive to each orientation, and that neurons within a hypercolumn are all-to-all coupled (Blasdel [29]). This coupling is usually assumed to be inhibitory. Thus, when considering V1, the columns in the Wilson networks correspond to hypercolumns where the attributes are the direction of a line field at a specified area in the visual field. However, V1 imaging also indicates a second kind of coupling, called *lateral coupling* that connects neurons in neighboring hypercolumns [26,29]. Moreover, the neurons that are most strongly laterally coupled are those that have the same orientation sensitivity [26,30], albeit at different points in the visual field. Finally, lateral coupling is usually taken to be excitatory. 

With the structure of V1 as inspiration, we define an excitatory lateral coupling in the Wilson networks by connecting those nodes in different columns that correspond to the *same level*. See Fig. 5(c).

**Fig. 6:**
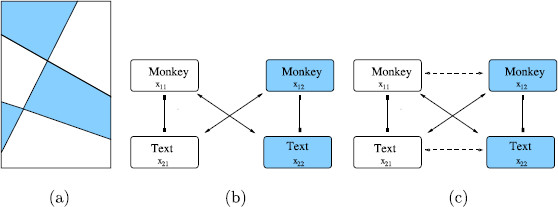
**a** Distinct areas in scrambled *monkey*-*text* experiment. **b** Schematic two-attribute two-pattern Wilson network for scrambled *monkey*-*text* experiment with reciprocal inhibition in attribute columns and reciprocal excitation in learned patterns. **c** Wilson network with reciprocal lateral excitation

The scrambled *monkey*-*text* experiment can be modeled by a two-level, two-attribute Wilson network with two learned patterns. To specify the network, we conceptualize the Kovács images in Fig. 3(b) as rectangles divided into two regions: one indicated by white and the other by blue in Fig. 6(a). The first attribute in the network corresponds to the portion of a rectangular image in the white region and the second attribute corresponds to the portion of that rectangular image in the blue region. In the Kovács experiment, the possible levels of each attribute are the portion of the *monkey* image in the associated region and the portion of the *text* image in that region. 

This network has four nodes, where $X_{ij}$ represents level *i* of attribute *j* as shown in Fig. 6(b). More specifically, $X_{11}$ represents *monkey* in the white region in Fig. 6(a) and $X_{21}$ represents *text* in the white region. Similarly, $X_{12}$ represents *monkey* in the blue region and $X_{22}$ represents *text* in the blue region. Thus, there are reciprocal inhibitory connections between nodes $X_{11}$ and $X_{21}$ and between $X_{12}$ and $X_{22}$. There are also reciprocal excitatory connections between $X_{11}$ and $X_{22}$ and between $X_{21}$ and $X_{12}$ representing the two learned patterns. In this network, the state {$x_{11}^{E}>x_{21}^{E}$ and $x_{22}^{E}>x_{12}^{E}$} corresponds to the scrambled image in Fig. 3(b)(left), whereas the state {$x_{21}^{E}>x_{11}^{E}$ and $x_{12}^{E}>x_{22}^{E}$} corresponds to the scrambled image in Fig. 3(b)(right). Importantly, the network also supports two derived pattern states: {$x_{11}^{E}>x_{21}^{E}$ and $x_{12}^{E}>x_{22}^{E}$}, which corresponds to the *monkey* only image in Fig. 3(a)(left), and {$x_{21}^{E}>x_{11}^{E}$ and $x_{22}^{E}>x_{12}^{E}$}, which corresponds to the *text* only image in Fig. 3(a)(right).

**Fig. 7:**
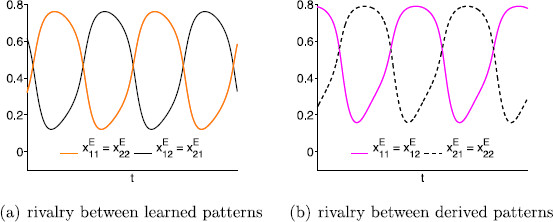
Simulations of network in Fig. 6(c) showing stable rivalry for equations in (11), where $G(z)=\frac{0.8}{1+e^{-7.2(z-0.9)}}$, I=2, w=0.25, β=1.5, g=1, ε=0.6667. In **a**, δ=0 and in **b**δ=0.5, where *δ* is the strength of the lateral coupling

Note that lateral coupling changes the network in Fig. 6(b) to the one in Fig. 6(c). Simulations of the equations associated with the network in Fig. 6(c) show stable rivalrous solutions between both learned and derived patterns (see Fig. 7). These simulations use the standard rate equations (11) introduced in Sect. 4. 

The symmetries of the networks in Fig. 6(b) and 6(c) are the same. Hence, for this experiment, the addition of lateral coupling does not change the expected types of periodic solutions that can be obtained through symmetry-breaking Hopf bifurcation. However, lateral coupling does change the symmetry of the Wilson network (and hence the expected types of solutions) corresponding to the scrambled *colored dot* experiment as shown in Fig. 9.

**Fig. 8:**
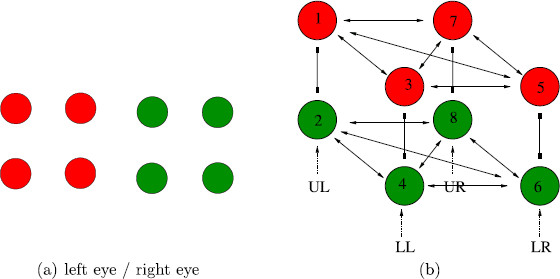
**a** Images in simplification of the conventional *colored dot* experiment in <abbrgrp>^2^,^25^</abbrgrp>. **b** Network with two learned patterns corresponding to the simplified experiment; symmetry group is $\Gamma =S_{4}\times Z_{2}(\rho )$. UL=upper left, LL=lower left, LR=lower right, UR=upper right

Tong et al. [25] suggest a simplified version of the *colored dot* experiments in [2], where each eye is presented with a square symmetric pattern of four dots. So, in the Tong version of the conventional *colored dot* experiment, one learned pattern has four red dots and the other has four green dots, as shown in Fig. 8(a). To our knowledge, this proposed rivalry experiment has not been performed. 

We model this experiment by a Wilson network consisting of four attribute columns, where each attribute refers to the position of one of the dots (upper left, lower left, lower right, upper right) and has two levels (red and green). The eight-node Wilson network with two learned patterns is shown in Fig. 8(b). Adding lateral coupling to this network does not change the symmetry since the lateral coupling and the learned pattern coupling are coincident in this model.

**Fig. 9:**
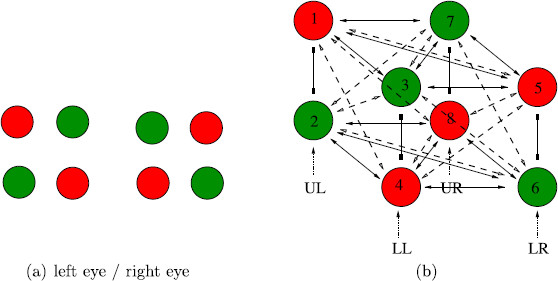
**a** Images in simplification of the scrambled *colored dot* experiment in <abbrgrp>^2^,^25^</abbrgrp>. **b** Network with two learned patterns and lateral coupling corresponding to the simplified experiment; symmetry group is $\Gamma =D_{4}\times Z_{2}(\rho )$

The Wilson network with lateral coupling for the scrambled *colored dot* experiment is shown in Fig. 9(b). This network is presented so that the learned pattern couplings are in horizontal planes; that is, the red and green levels are inverted in the LL and UR attribute columns. Note that if lateral coupling were not included then this network would be isomorphic to Fig. 8(b), and hence have the same symmetry groups. It would follow that we would predict the same solution types for the two *colored dot* experiments, which is not what is observed. 

## 3 Symmetry and Hopf Bifurcation

Wilson networks have symmetry and these symmetries dictate the kinds of periodic solutions that can be obtained through Hopf bifurcation from a fusion state. The classification of periodic solutions proceeds as follows. See [5]. 

(1) Determine the symmetry group *Γ* of the network and how *Γ* acts on phase space.

(2) Determine the irreducible representations of this action of *Γ*. (Recall that a *representationV* is an invariant subspace of the action of *Γ*; *V* is *irreducible* if the only invariant subspaces are the trivial subspace {0} and *V* itself.)

(3) Classify the periodic solutions for each distinct irreducible representation by their spatiotemporal symmetries.

 Step 1 is straightforward for the networks we consider. Step 2 is most easily determined by computing the isotypic decomposition of *Γ*. An *isotypic component* consists of the sum of all isomorphic irreducible representations. In general, step 3 is difficult, but it has been worked out in the literature for most standard group actions. Note that if a symmetry γ∈Γ acts trivially on an isotypic component, then all bifurcating periodic solutions corresponding to this component will be invariant under the symmetry. This remark enables us to identify representations that only lead to oscillating fusion states, which are uninteresting from the rivalry point of view. Specifically, let *ρ* be the symmetry that transposes the two nodes in each column. A solution that is invariant under *ρ* will have activity variables equal in each column and, therefore, be fusion states.

### 3.1 The Scrambled *Monkey*-*Text* Experiment Networks

The form of equations relevant to the network in Fig. 6(b) is

(2) $$\begin{array}{rl} {\dot{X}}_{11} & =F(X_{11},X_{21},X_{22})\\ {\dot{X}}_{21} & =F(X_{21},X_{11},X_{12})\\ {\dot{X}}_{12} & =F(X_{12},X_{22},X_{21})\\ {\dot{X}}_{22} & =F(X_{22},X_{12},X_{11}) \end{array}$$

where in F(X,Y,Z), *X* is the internal state variable of the given node, *Y* is the node connected to *X* with inhibitory coupling, and *Z* is the node connected to *X* with excitatory learned pattern coupling. Note that for general networks $X_{ij}\in R^{k}$. However, in the models we use, k=2.

We claim that two types of non-fusion oscillation can be obtained by Hopf bifurcation from fusion states ($X_{11}=X_{12}=X_{21}=X_{22}$). Our argument is based on symmetry and utilizes the theory of Hopf bifurcation in the presence of symmetry [5]. The symmetry group $D_{2}$ of this Wilson network is generated by two symmetries, namely, the symmetry *ρ* that swaps rows and the symmetry *κ* that swaps columns. Specifically,

$$\begin{array}{rl} \rho (X_{11},X_{21},X_{12},X_{22}) & =(X_{21},X_{11},X_{22},X_{12})\\ \kappa (X_{11},X_{21},X_{12},X_{22}) & =(X_{12},X_{22},X_{11},X_{21}) \end{array}$$

An important consequence of symmetry is that at a symmetric equilibrium the Jacobian of a symmetric system of differential equations, such as (2), is block diagonalized by the isotypic decomposition of the symmetry group acting on phase space [5]. 

The isotypic decomposition for $D_{2}$ on $R^{8}$ is given by

(3) $$R^{8}=V^{++}⊕V^{+-}⊕V^{-+}⊕V^{--}$$

where the $V^{ab}$ are defined in (4).

(4) $$\begin{array}{rl} V^{++} & =\left\{(X,X,X,X)\right\}\;\rho =1,\kappa =1\;\text{fusion}\\ V^{+-} & =\left\{(X,X,-X,-X)\right\}\;\rho =1,\kappa =-1\;\text{fusion}\\ V^{-+} & =\left\{(X,-X,X,-X)\right\}\;\rho =-1,\kappa =1\;\text{derived: unscrambled}\\ V^{--} & =\left\{(X,-X,-X,X)\right\}\;\rho =-1,\kappa =-1\;\text{learned: scrambled} \end{array}$$

where $X=(x^{E},x^{H})\in R^{2}$. Note that any point $(X_{11},X_{21},X_{12},X_{22})\in R^{8}$ that is fixed by *ρ* satisfies $X_{11}=X_{21}$ and $X_{12}=X_{22}$. Since the attribute levels of such states are equal, these states are fusion states and are so labeled in (4). It also follows from the theory of Hopf bifurcation with symmetry and from (4) that Eq. (2) have four possible types of Hopf bifurcation from a fusion state *X* where all $X_{ij}$ are equal. One type of bifurcation leads to rivalry between learned patterns, a second type leads to rivalry between derived patterns, and as noted the remaining two types (where *ρ* fixes all points in the isotypic component) lead to rivalry between fusion states.

### 3.2 The Conventional *Colored Dot* Network

The Wilson network in Fig. 8(b) has $S_{4}\times Z_{2}(\rho )$ symmetry, where $S_{4}$ is the permutation group of the four attribute columns and $Z_{2}(\rho )$ interchanges the upper and lower nodes in each column. The rivalry predictions from this network require using the theory of Hopf bifurcation in the presence of $S_{4}$ symmetry (Stewart [27] and Dias and Rodrigues [31]). 

Equivariant Hopf bifurcation is driven by the irreducible representations of $\Gamma =S_{4}\times Z_{2}(\rho )$ on $R^{8}$ and there are four such distinct irreducible representations. First, recall that $S_{4}$ decomposes $R^{4}$ into two (absolutely) irreducible representations

$$\begin{array}{rcl} V_{1} & = & \left\{(X,X,X,X):X\in R^{2}\right\}\\ V_{3} & = & \left\{(X_{1},X_{2},X_{3},X_{4}):X_{j}\in R^{2};X_{1}+X_{2}+X_{3}+X_{4}=0\right\} \end{array}$$

It follows that the irreducible representations of *Γ* acting on $R^{8}=R^{4}⊕R^{4}$ are

(5) $$\begin{array}{rl} V_{1}^{+} & =\left\{(v,v):v\in V_{1}\right\}\;\text{fusion}\\ V_{1}^{-} & =\left\{(v,-v):v\in V_{1}\right\}\;\text{learned: single color}\\ V_{3}^{+} & =\left\{(v,v):v\in V_{3}\right\}\;\text{fusion}\\ V_{3}^{-} & =\left\{(v,-v):v\in V_{3}\right\}\;\text{derived: scrambled colors} \end{array}$$

The decomposition (5) is the analog for the conventional *colored dot* network of the decomposition (4) for the scrambled *monkey*-*text* network. Note that *ρ* acts trivially in the plus representations and as multiplication by −1 in the minus representations. All solutions bifurcating from a plus representation are invariant under *ρ*, and hence are fusion states, since invariance under *ρ* implies that the entries in each attribute column are equal.

On the other hand, all periodic solutions bifurcating from a minus representation satisfy

(6) $$X(t)=\left(\begin{array}{cccc} a_{0} & b_{0} & c_{0} & d_{0}\\ a_{1/2} & b_{1/2} & c_{1/2} & d_{1/2} \end{array}\right)$$

We use the notation $e_{\theta }(t)=e(t+\theta T)$ where e(t) is *T*-periodic. Hopf bifurcation based on $V_{1}^{-}$ leads to solutions of the form of $\Sigma_{0}$ in Table 1, that is, to rivalry between the two learned patterns in Fig. 8(a). 

**Table 1:** Isotropy subgroups of periodic solutions from $S_{4}\times Z_{2}(\rho )$ symmetry. We use the notation $e_{\theta }(t)=e(t+\theta T)$ where e(t) is *T*-periodic. Moreover, the frequency of *u* is three times the frequency of *a*, v≈−3a, and the frequency of *c* is twice the frequency of *a*

*Σ*	Pattern of oscillation *X*(*t*)	
$\Sigma_{0}$	$\left(\begin{array}{cccc} a_{0} & a_{0} & a_{0} & a_{0}\\ a_{1/2} & a_{1/2} & a_{1/2} & a_{1/2} \end{array}\right)$	Figure 8(a)
$\Sigma_{1}$	$\left(\begin{array}{cccc} a_{0} & a_{1/2} & a_{1/2} & a_{0}\\ a_{1/2} & a_{0} & a_{0} & a_{1/2} \end{array}\right)$	Figures 10(a) or 10(b) or 10(c)
$\Sigma_{2}$	$\left(\begin{array}{cccc} c & a_{0} & a_{1/2} & c\\ c & a_{1/2} & a_{0} & c \end{array}\right)$	Fusion
$\Sigma_{3}$	$\left(\begin{array}{cccc} a_{0} & a_{1/4} & a_{2/4} & a_{3/4}\\ a_{2/4} & a_{3/4} & a_{0} & a_{1/4} \end{array}\right)$	Figure 11
$\Sigma_{4}$	$\left(\begin{array}{cccc} a_{0} & a_{2/6} & a_{4/6} & u_{0}\\ a_{3/6} & a_{5/6} & a_{1/6} & u_{1/2} \end{array}\right)$	Complicated transitions
$\Sigma_{5}$	$\left(\begin{array}{cccc} a_{0} & a_{0} & a_{0} & v_{0}\\ a_{1/2} & a_{1/2} & a_{1/2} & v_{1/2} \end{array}\right)$	Figure 12

**Fig. 10:**
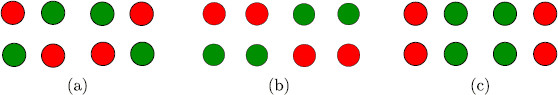
Predicted percept alternations for proposed conventional *colored dot* experiment. Rivalry between *two red* and *two green dot* patterns: **a** diagonal; **b** adjacent top and bottom; **c** adjacent sides

**Fig. 11:**
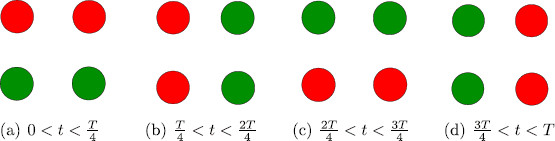
Predicted percept alternations for proposed conventional *colored dot* experiment. Rivalry in a rotating wave

**Fig. 12:**
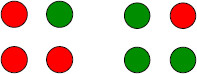
Predicted percept alternations for proposed conventional *colored dot* experiment. Rivalry between *three dots of one color*

Next, we consider Hopf bifurcation based on $V_{3}^{-}$. This bifurcation is driven by Hopf bifurcation of $S_{4}$ on $V_{3}$, which has been analyzed in [27]. (The stability of resulting solutions is discussed on p. 634 in [31].) Up to conjugacy, these authors find five types $\Sigma_{1},\ldots ,\Sigma_{5}$ of periodic solutions whose structures are listed in Table 1. Patterns corresponding to $\Sigma_{1}$ give rivalry between the derived patterns shown in Fig. 10(a) (note that because of symmetry, Figs. 10(b) and 10(c) are conjugate to Fig. 10(a), and all three patterns coexist). Patterns corresponding to $\Sigma_{3}$ are those shown in Fig. 11; patterns corresponding to $\Sigma_{5}$ are those shown in Fig. 12. We have not computed the transition of patterns that are associated with $\Sigma_{4}$ solutions. 

We have focused on the simplified version of the conventional *colored dot* experiment with a 2×2 grid of dots. However, the bifurcations using a 6×4 grid of dots, as in the original experiment [2], are completely analogous. Suppose there are *n* dots. Then there will be *n* attribute columns with a symmetry group $\Gamma =S_{n}\times Z_{2}(\rho )$. The isotypic decomposition is

$$\begin{array}{rl} V_{1} & =\left\{(X,\ldots ,X):X\in R^{2}\right\}\\ V_{n-1} & =\left\{(X_{1},\ldots ,X_{n}):X_{j}\in R^{2};X_{1}+\cdots +X_{n}=0\right\} \end{array}$$

It follows that the irreducible representations of *Γ* acting on $R^{2n}=R^{n}⊕R^{n}$ are

(7) $$\begin{array}{rl} V_{1}^{+} & =\left\{(v,v):v\in V_{1}\right\}\;\text{fusion}\\ V_{1}^{-} & =\left\{(v,-v):v\in V_{1}\right\}\;\text{learned: single color}\\ V_{n-1}^{+} & =\left\{(v,v):v\in V_{n-1}\right\}\;\text{fusion}\\ V_{n-1}^{-} & =\left\{(v,-v):v\in V_{n-1}\right\}\;\text{derived: scrambled colors} \end{array}$$

Hence, the bifurcation structure for *n* dots is analogous to that of 4 dots; there are two types of bifurcation to fusion states ($V_{n-1}^{+}$, $V_{1}^{+}$), one to rivalry between the learned patterns ($V_{1}^{-}$), and one to bifurcation to derived patterns ($V_{n-1}^{-}$). The actual solution types depend on *n* and we will not attempt to interpret the bifurcation results of [27] in the *n* dot case as we have in the four dot case.

### 3.3 The Scrambled *Colored Dot* Network

Next, we return to the Kovács scrambled *colored dot* experiment where the subjects are shown the scrambled colored images in Fig. 4(b). In this case, subjects report perceiving rivalry between the all red dot and all green dot images in Fig. 4(a) for nearly 50 % of the duration of the experiment. This result is difficult to explain with a standard Wilson network. The reason is that when lateral coupling is ignored, this experiment leads to a Wilson network with the same symmetry group as the conventional Kovács dot experiment. It follows that rivalry between the images in Fig. 4(a) is one of several types of possible solutions and it is not clear why this particular solution type should be observed for such a large percentage of the time.

If, however, we include lateral coupling we arrive at the network in Fig. 9 whose symmetry group is $\Gamma =D_{4}\times Z_{2}(\rho )$. Differential equations that correspond to this network have the form 

 where the overbar indicates terms whose order can be interchanged. The form of (8) emphasizes the fact that there are three different types of coupling: inhibitory, excitatory learned, and excitatory lateral.

The isotypic decomposition of $R^{8}$ under $\Gamma =D_{4}\times Z_{2}(\rho )$ now has six components, as follows. Let

$$\begin{array}{rcl} W_{0} & = & \left\{(X,X,X,X):X\in R^{2}\right\}\\ W_{1} & = & \left\{(X,-X,X,-X):X\in R^{2}\right\}\\ W_{2} & = & \left\{(X_{1},X_{2},-X_{1},-X_{2}):X_{1},X_{2}\in R^{2}\right\} \end{array}$$

It follows that the isotypic components of *Γ* acting on $R^{16}=R^{8}⊕R^{8}$ are

(9) $$\begin{array}{rl} W_{0}^{+} & =\left\{(v,v):v\in W_{0}\right\}\;\text{fusion}\\ W_{1}^{+} & =\left\{(v,v):v\in W_{1}\right\}\;\text{fusion}\\ W_{2}^{+} & =\left\{(v,v):v\in W_{2}\right\}\;\text{fusion}\\ W_{0}^{-} & =\left\{(v,-v):v\in W_{0}\right\}\;\text{learned: scrambled color}\\ W_{1}^{-} & =\left\{(v,-v):v\in W_{1}\right\}\;\text{derived: single color}\\ W_{2}^{-} & =\left\{(v,-v):v\in W_{2}\right\}\;\text{derived: other scrambled colors} \end{array}$$

As in the previous examples, bifurcation with respect to $W_{j}^{+}$ leads to fusion states. Bifurcations with respect to $W_{0}^{-}$ leads to rivalry between the learned patterns and bifurcation with respect to $W_{1}^{-}$ leads to rivalry between the single color dots, as desired. Finally, bifurcation with respect to $W_{2}^{-}$ leads to scrambled color patterns similar to (but not the same as) those obtained in the $S_{4}\times Z_{2}(\rho )$ case. From an abstract point of view, the Wilson network with lateral coupling is a much more satisfactory explanation for the existence of the single color rivalry when scrambled dots are presented than is the Wilson network without lateral coupling.

Finally, we note that the discussion in this section generalizes to the scrambled dot experiment with a 6×4 grid of colored dots, as long as the number of green dots and the number of red dots in the scrambled learned patterns are equal, as in Fig. 4(b).

## 4 Stability in Scrambled *Monkey*-*Text* Networks

The classification of possible solution types, as given in Sect. 3, is model independent. We do not need to know the particular equations in order to complete the classification; we just need to know that the equations are *Γ*-equivariant. Given a system of equations, we can prove that solutions of the types that we have classified actually exist only by showing that a Hopf bifurcation that corresponds to the appropriate isotypic component actually occurs. See the equivariant Hopf theorem in [5]. We can also determine whether these solutions are stable, which is model dependent; we need to know the equations. 

There are three steps in the calculation of stability. First, we need to determine that there is a fusion equilibrium. Second, we must show that the Hopf bifurcations themselves can be stable. That is, we must find Hopf bifurcation points where the critical eigenvectors of the Jacobian *J* at the fusion equilibium correspond to the given isotypic component and all other eigenvalues of *J* have negative real part. Third, we need to calculate higher order terms in a center manifold reduction to check that the bifurcating solutions are actually stable. Alternatively, we can just simulate the equations for parameter values near a stable Hopf point and see whether we can detect stable solutions. Indeed, this was our approach for the scrambled *monkey-text* model in Sect. 2.

The principal conclusion is that derived pattern rivalry (between unscrambled images) can be stable in this model only if the strength of the lateral coupling is greater than the strength of the learned pattern coupling (see Proposition 3). Note that this cannot happen if lateral coupling is absent. We also show that learned pattern rivalry (between scrambled images) can only be stable when the strength of the learned pattern coupling is greater than the strength of the lateral coupling (see Proposition 2).

### 4.1 Equations for the Scrambled *Monkey*-*Text* Network

There is some leeway in choosing differential equations associated to a given network. In this context, we follow Wilson and others and assume that the nodes are neurons or groups of neurons and that the important information is captured by the firing rate of the neurons. Thus, we follow [1] and assume that in these models each node (i,j) in the network has a state space $x_{ij}=(x_{ij}^{E},x_{ij}^{H})$, where $x_{ij}^{E}$ is an activity variable (representing firing rate) and $x_{ij}^{H}$ is a fatigue variable. Coupling between nodes is given through a gain function G. Specifically,

(10) $$\begin{array}{rl} \varepsilon {\dot{x}}_{ij}^{E} & =-x_{ij}^{E}+G\left(I_{ij}+w\sum_{pq\to ij}x_{pq}^{E}+\delta \sum_{uv\mapsto ij}x_{uv}^{E}-\beta \sum_{rj\Rightarrow ij}x_{rj}^{E}-gx_{ij}^{H}\right)\\ {\dot{x}}_{ij}^{H} & =x_{ij}^{E}-x_{ij}^{H} \end{array}$$

where → indicates an excitatory learned pattern connection, ↦ indicates an excitatory lateral connection, and ⇒ indicates an inhibitory connection. Similar rate models are often used in the rivalry literature (Wilson et al. [32,33]). The parameters are: reciprocal learned pattern excitation between nodes w>0, reciprocal lateral excitation δ≥0, reciprocal inhibition between nodes β>0, the external signal strength $I_{ij}\geq 0$ to nodes, the strength of reduction of the activity variable by the fatigue variable g>0, and the ratio of time scales ε<1 on which ${*}^{E}$ and ${*}^{H}$ evolve. Note that δ=0 for the simulations in [1]. The gain function G is usually assumed to be nonnegative and nondecreasing, and is often a sigmoid.

In this case, we assume all $I_{ij}=I$ and for the network in Fig. 6(c) the system (10) reduces to:

(11) $$\begin{array}{rl} \varepsilon {\dot{x}}_{11}^{E} & =-x_{11}^{E}+G\left(I+wx_{22}^{E}+\delta x_{12}^{E}-\beta x_{21}^{E}-gx_{11}^{H}\right)\\ {\dot{x}}_{11}^{H} & =x_{11}^{E}-x_{11}^{H}\\ \varepsilon {\dot{x}}_{21}^{E} & =-x_{21}^{E}+G\left(I+wx_{12}^{E}+\delta x_{22}^{E}-\beta x_{11}^{E}-gx_{21}^{H}\right)\\ {\dot{x}}_{21}^{H} & =x_{21}^{E}-x_{21}^{H}\\ \varepsilon {\dot{x}}_{12}^{E} & =-x_{12}^{E}+G\left(I+wx_{21}^{E}+\delta x_{11}^{E}-\beta x_{22}^{E}-gx_{12}^{H}\right)\\ {\dot{x}}_{12}^{H} & =x_{12}^{E}-x_{12}^{H}\\ \varepsilon {\dot{x}}_{22}^{E} & =-x_{22}^{E}+G\left(I+wx_{11}^{E}+\delta x_{21}^{E}-\beta x_{12}^{E}-gx_{22}^{H}\right)\\ {\dot{x}}_{22}^{H} & =x_{22}^{E}-x_{22}^{H} \end{array}$$

As we will see, there is an advantage of lateral coupling in the four-node model for the scrambled *monkey*-*text* experiment. The additional coupling allows the rivalrous solutions with respect to the derived patterns to be asymptotically stable at bifurcation; these solutions are not stable if lateral coupling is excluded.

### 4.2 Calculation of Fusion Equilibria

The equations for a fusion equilibrium for (11) reduces to

(12) x=G(I+(w+δ−β−g)x)

where all $x_{ij}^{E}=x_{ij}^{H}=x$. Solutions of this equation have been studied by [10,11,34]. It is convenient to define

ρ=w+δ−β−g

Then (12) can be rephrased as

(13) G(I+ρx)−x=0

Diekman et al. (Lemma 3.1 in [34]) state that for every *ρ* there is an I>0 and x>0 that satisfies (13). Thus, we can assume there is a fusion state for any choice of *w*, *δ*, *β*, *g*, *ε*. We are particularly interested in the case when ρ<0.

**Lemma 1***Fix*$w,\delta ,\beta ,g,I,G_{0}^{\prime }>0$. *Fix*$x_{0}>0$*so that the*$(I-x_{0})\rho >0$. *Then there exists a gain function*G(z)*satisfying*

(14) $$G(x_{0})=\frac{1}{\rho }(x_{0}-I)$$

*and*$G^{\prime }(z_{0})=G_{0}^{\prime }$.

It follows from Lemma 1 that $x_{0}$ is a fusion equilibrium and that we can choose $G^{\prime }(x_{0})>0$ arbitrarily.

*Proof of Lemma 1* The sigmoidal function

$$G(x)=\frac{2a}{1+e^{-(2b/a)(x-x_{0})}}$$

satisfies $G(x_{0})=a$ and $G^{\prime }(x_{0})=b$. Set $b=G_{0}^{\prime }>0$ and *a* equal to the RHS of (14), which is also positive since *ρ* and $x_{0}-I$ have the same sign. □

### 4.3 Calculation of Critical Eigenvalues

(15) $$\begin{array}{rl} J^{++} & =\left(\begin{array}{cc} -1+(w+\delta -\beta )G^{\prime } & -gG^{\prime }\\ \varepsilon & -\varepsilon \end{array}\right)\\ J^{+-} & =\left(\begin{array}{cc} -1+(-w-\delta -\beta )G^{\prime } & -gG^{\prime }\\ \varepsilon & -\varepsilon \end{array}\right)\\ J^{-+} & =\left(\begin{array}{cc} -1+(-w+\delta +\beta )G^{\prime } & -gG^{\prime }\\ \varepsilon & -\varepsilon \end{array}\right)\\ J^{--} & =\left(\begin{array}{cc} -1+(w-\delta +\beta )G^{\prime } & -gG^{\prime }\\ \varepsilon & -\varepsilon \end{array}\right) \end{array}$$

(16) $$\begin{array}{rl} \det\left(J^{++}\right) & =\varepsilon \left(1+(g+\beta -w-\delta )G^{\prime }\right)\\ \det\left(J^{+-}\right) & =\varepsilon \left(1+(g+\beta +w+\delta )G^{\prime }\right)\\ \det\left(J^{-+}\right) & =\varepsilon \left(1+(g-\beta +w-\delta )G^{\prime }\right)\\ \det\left(J^{--}\right) & =\varepsilon \left(1+(g-\beta -w+\delta )G^{\prime }\right) \end{array}$$

(17) $$\begin{array}{rl} \mathrm{tr}\left(J^{++}\right) & =-1+(w+\delta -\beta )G^{\prime }-\varepsilon \\ \mathrm{tr}\left(J^{+-}\right) & =-1+(-w-\delta -\beta )G^{\prime }-\varepsilon \\ \mathrm{tr}\left(J^{-+}\right) & =-1+(-w+\delta +\beta )G^{\prime }-\varepsilon \\ \mathrm{tr}\left(J^{--}\right) & =-1+(w-\delta +\beta )G^{\prime }-\varepsilon \end{array}$$

For Hopf bifurcation to exist, we need one trace to be zero and the corresponding determinant to be positive. For that Hopf bifurcation to be stable, we require all four determinants to be positive and the remaining three traces to be negative.

### 4.4 Stability of Learned Pattern Rivalry

**Proposition 2***To have stable Hopf bifurcation to learned pattern rivalry in* (11), *it is necessary that*

(18) $$\begin{array}{rl} \beta & >\delta \\ w & >\delta \end{array}$$

*Sufficient conditions for stable Hopf bifurcation to learned pattern rivalry are given by* (18) *and*

(19) $$\begin{array}{rl} g & >w-\delta +\beta \\ G^{\prime } & >\frac{1}{w-\delta +\beta } \end{array}$$

*Proof* For Hopf bifurcation to learned pattern rivalry to exist, we need $\mathrm{tr}(J^{--})=0$, that is,

$$\varepsilon =-1+(w-\delta +\beta )G^{\prime }$$

It follows from (19) that ε>0. For this bifurcation to be stable we also need the other three traces to be negative. Thus, substituting for *ε* in (17), we obtain the necessary conditions

(20) $$\begin{array}{rl} \mathrm{tr}\left(J^{++}\right) & <0\;\beta >\delta \\ \mathrm{tr}\left(J^{+-}\right) & <0\;\beta +w>0\\ \mathrm{tr}\left(J^{-+}\right) & <0\;w>\delta \end{array}$$

Note that the necessary conditions (18) follow from directly from (20) and the second condition in (20) follows from the first and third.

To prove the sufficiency part of the lemma, we need to verify that the determinants are all positive. This follows from (16) if

(21) $$\begin{array}{rl} g+\beta -w-\delta & >0\\ g+\beta +w+\delta & >0\\ g-\beta +w-\delta & >0\\ g-\beta -w+\delta & >0 \end{array}$$

Note that the second inequality is always satisfied and, assuming (18), the first and third follow from the fourth. Finally, the fourth inequality follows from (19). □

Note that Hopf bifurcation to stable learned patterns is possible when the lateral coupling is nonexistent; that is, δ=0.

### 4.5 Stability of Derived Pattern Rivalry

**Proposition 3***To have stable Hopf bifurcation to learned pattern rivalry in* (11), *it is necessary that*

(22) $$\begin{array}{rl} \beta & >w\\ \delta & >w \end{array}$$

*Sufficient conditions for stable Hopf bifurcation to learned pattern rivalry are given by* (22) *and*

(23) $$\begin{array}{rl} g & >\delta -w+\beta \\ G^{\prime } & >\frac{1}{\delta -w+\beta } \end{array}$$

*Proof* For Hopf bifurcation to derived pattern rivalry, we need $\mathrm{tr}(J^{-+})=0$, that is,

$$\varepsilon =-1+(-w+\delta +\beta )G^{\prime }$$

It follows from (23) that ε>0. For this bifurcation to be stable, we need the other three traces to be negative. On substituting for *ε* in (17), we obtain the necessary conditions:

(24) $$\begin{array}{rl} \mathrm{tr}\left(J^{++}\right) & <0\;\beta >w\\ \mathrm{tr}\left(J^{+-}\right) & <0\;\beta +\delta >0\\ \mathrm{tr}\left(J^{--}\right) & <0\;\delta >w \end{array}$$

Note that the necessary conditions (22) follow directly from (24) and the second condition in (24) follows from the first and third.

To prove the sufficiency part of the lemma, we need to verify that the determinants are all positive. This follows from (16) if the four conditions (21) are satisfied. Note that the second inequality is always satisfied and, assuming (22), the first and fourth follow from the third. Finally, the third inequality follows from (23). □

Note that Hopf bifurcation to stable derived patterns is possible only when the strength of the lateral coupling is larger than the strength of the learned pattern coupling; that is, δ>w.

## 5 Discussion

We have shown that the surprising results in three binocular rivalry experiments described by Kovács et al. [2] can be understood through the use of Wilson-type networks [1] and equivariant Hopf bifurcation theory [5], as interpreted in coupled cell systems [3]. 

We would like to put our results in a broader context. We showed in Diekman et al. [34] that rivalry between two patterns in Wilson networks collapses to the two-node network in Fig. 2 when the patterns have no attribute levels in common. This reduction uses the notion of a quotient network discussed in [3] and proceeds by identifying equivalent levels in different attribute columns. Let S denote the subspace obtained in this way [34]. This subspace is flow-invariant for the dynamics; moreover, if one uses the rate models (10) (without lateral coupling), then there are regions in parameter space where the dynamics are attracting to S. We mention this for two reasons. First, bifurcation in directions transverse to S yields the derived patterns discussed in this paper. For such bifurcations to occur, S cannot be attracting and this occurs when lateral coupling is present. Second, one can think of the reduction to S (that is, reduction to the two-node network) as aggregating the information contained in several different attributes into one combined attribute. We believe this is a more general phenomenon with different levels of pattern complexity, as we now describe.

To construct a Wilson network for a given experiment, we must assume which attributes and which levels appropriately define a pattern. For example, in the simplified *colored dot* experiments, we assume that the attributes are the colors of the dots at four geometric locations. On the other hand, in the scrambled *monkey*-*text* experiment, we assume that the attributes are the kind of picture (monkey or text) in two regions of the image rectangle (the blue and the white regions in Fig. 6(a)). One can ask whether these attributes are the reasonable ones to describe patterns in these experiments.

**Fig. 13:**
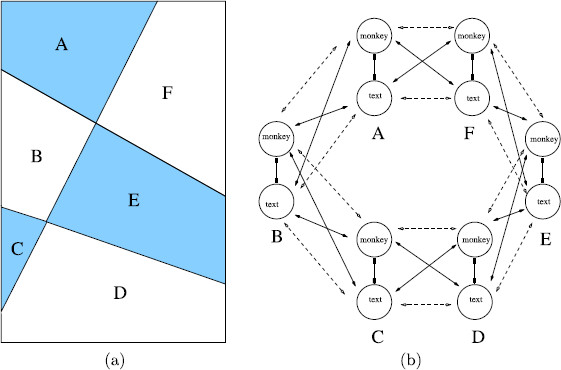
**a***Regions A–F* in *image rectangle* of scrambled *monkey*-*text* experiment. **b** Network with *six attribute columns* corresponding to monkey or text image in each region. All inhibitory couplings are shown, but only “nearest neighbor” learned and lateral couplings are shown

For example, suppose we assume that the attributes in the scrambled *monkey*-*text* experiment are the type of image in the six regions labeled A–F in Fig. 13(a). Then we are led to the 12-node network in Fig. 13(b) as a model for this experiment. Such a decomposition is closer in spirit to the geometric decomposition in the *colored dot* experiments. It is reasonable to ask whether there is a relationship between the networks in Figs. 6(c) and 13(b), and there is. The larger network in Fig. 13(b) has a quotient network on the flow-invariant subspace

$$\mathrm{Fix}\left((\mathrm{ACE})(\mathrm{BDF})\right)=\{X_{iA}=X_{iC}=X_{iE}\text{ and }X_{iB}=X_{iD}=X_{iF}\text{ for }i=1,2\}$$

(see [3]) that is isomorphic to the smaller network in Fig. 6(c). Hence, the solution types that we discussed previously for the smaller network also appear in the larger network (which corresponds to a more refined geometry). In principle, other solution types can appear in the larger network, but there were no indications of such solutions in the scrambled *monkey*-*text* experiment. We believe that there is a general relationship between refined patterns (the addition of extra attribute columns in Wilson networks) and the quotient networks from coupled cell theory [3]. 

There are two prevalent views about what leads to alternations during binocular rivalry: *eye-based* theories postulate that the two eyes compete for dominance, while *stimulus-based* theories postulate that it is coherent perceptual representations that are in competition (Papathomas et al. [24]). Kovács et al. [2] interpreted their results on interocular grouping (IOG) as evidence against eye-based theories of rivalry. 

Lee and Blake [21] reexamine IOG during rivalry, and argue that, whereas IOG rules out models of rivalry in which one eye or the other is completely dominant at any given moment, IOG can be explained by simultaneous dominance of local eye-based regions distributed between the eyes. To demonstrate this, they performed a series of experiments using the Kovács *monkey-text* images and an *eye-swap* technique that exchanges rival images immediately after one becomes dominant (Blake et al. [35]). In their analysis, [21] consider a decomposition of the *monkey-text* images into six regions that is very similar to the decomposition shown in Fig. 13(a). Our mathematical construction, based on Wilson networks and an abstract notion of quotient networks, is not meant to represent V1 or any specific brain area. However, our results support the conclusion of [21] that global IOG (derived patterns) can be achieved by simultaneous local eye dominance. 

We end by noting that it should be possible to test our predictions of likely percepts by performing the simplified *colored dot* experiments. We also note that illusions are part of this network theory and they themselves can lead to interesting kinds of perceptual alternations. This topic, as well as symmetry-breaking steady-state bifurcations that lead to various types of winner-take-all states, will be discussed in future work.

## Competing Interests

The authors declare that they have no competing interests.

## Authors’ Contributions

All authors performed research. COD and MG drafted the manuscript. All authors read and approved the final manuscript.

## References

[B1] Wilson HR Jenkins M, Harris L Requirements for conscious visual processing Cortical Mechanisms of Vision 2009 Cambridge University Press, Cambridge 399 417

[B2] Kovács I Papathomas TV Yang M Fehér A When the brain changes its mind: interocular grouping during binocular rivalry Proc Natl Acad Sci USA 1996 3 15508 15511 10.1073/pnas.93.26.15508 8986842 PMC26435

[B3] Golubitsky M Stewart I Nonlinear dynamics of networks: the groupoid formalism Bull Am Math Soc 2006 3 305 364 10.1090/S0273-0979-06-01108-6

[B4] Golubitsky M Stewart I The Symmetry Perspective: From Equilibrium to Chaos in Phase Space and Physical Space 2002 Birkhäuser, Basel

[B5] Golubitsky M Stewart I Schaeffer DG Singularities and Groups in Bifurcation Theory: Volume II Applied Mathematical Sciences 69 1988 Springer, New York

[B6] Blake R Logothetis NK Visual competition Nat Rev, Neurosci 2002 3 1 11 10.1038/nrn701 11823801

[B7] **Your amazing brain** [http://www.youramazingbrain.org/supersenses/necker.htm]

[B8] Laing CR Frewen T Kevrekidis IG Reduced models for binocular rivalry J Comput Neurosci 2010 3 459 476 10.1007/s10827-010-0227-6 20182782

[B9] Moreno-Bote R Rinzel J Rubin N Noise-induced alternations in an attractor network model of perceptual bistability J Neurophysiol 2007 3 1125 1139 10.1152/jn.00116.2007 17615138 PMC2702529

[B10] Curtu R Singular Hopf bifurcations and mixed-mode oscillations in a two-cell inhibitory neural network Physica D 2010 3 504 514 10.1016/j.physd.2009.12.010

[B11] Curtu R Shpiro A Rubin N Rinzel J Mechanisms for frequency control in neuronal competition models SIAM J Appl Dyn Syst 2008 3 609 649 10.1137/070705842 20953287 PMC2954747

[B12] Kalarickal GJ Marshall JA Neural model of temporal and stochastic properties of binocular rivalry Neurocomputing 2000 3 843 853

[B13] Laing C Chow C A spiking neuron model for binocular rivalry J Comput Neurosci 2002 3 39 53 10.1023/A:1014942129705 11932559

[B14] Lehky SR An astable multivibrator model of binocular rivalry Perception 1988 3 215 228 10.1068/p170215 3067209

[B15] Matsuoka K The dynamic model of binocular rivalry Biol Cybern 1984 3 201 208 10.1007/BF00334466 6704442

[B16] Mueller TJ A physiological model of binocular rivalry Vis Neurosci 1990 3 63 73 10.1017/S0952523800002777 2265146

[B17] Noest AJ van Ee R Nijs MM van Wezel RJA Percept-choice sequences driven by interrupted ambiguous stimuli: a low-level neural model J Vis 2007 3 8 Article ID 10 10.1167/7.8.10 17685817

[B18] Seely J Chow CC The role of mutual inhibition in binocular rivalry J Neurophysiol 2011 3 2136 2150 10.1152/jn.00228.2011 21775721 PMC3296268

[B19] Liu L Tyler CW Schor CM Failure of rivalry at low contrast: evidence of a suprathreshold binocular summation process Vis Res 1992 3 1471 1479 10.1016/0042-6989(92)90203-U 1455720

[B20] Shpiro A Curtu R Rinzel J Rubin N Dynamical characteristics common to neuronal competition models J Neurophysiol 2007 3 462 473 10.1152/jn.00604.2006 17065254 PMC2702527

[B21] Lee S Blake R A fresh look at interocular grouping during binocular rivalry Vis Res 2004 3 983 991 10.1016/j.visres.2003.12.007 15031091

[B22] Diaz-Caneja E: **Sur l’alternance binoculaire**. *Ann Ocul* 1928, **October**:721-731.

[B23] Alais D O’Shea RP Mesana-Alais C Wilson IG On binocular alternation Perception 2000 3 1437 1445 10.1068/p3017 11257967

[B24] Papathomas TV Kovács I Conway T Alais D, Blake R Interocular grouping in binocular rivalry: basic attributes and combinations Binocular Rivalry 2005 MIT Press, Cambridge 155 168

[B25] Tong F Meng M Blake R Neural bases of binocular rivalry Trends Cogn Sci 2006 3 502 511 10.1016/j.tics.2006.09.003 16997612

[B26] Bressloff PC Cowan JD Golubitsky M Thomas PJ Wiener MC Geometric visual hallucinations, Euclidean symmetry, and the functional architecture of striate cortex Philos Trans R Soc Lond B, Biol Sci 2001 3 299 330 10.1098/rstb.2000.0769 11316482 PMC1088430

[B27] Stewart I Symmetry methods in collisionless many-body problems J Nonlinear Sci 1996 3 543 563 10.1007/BF02434056

[B28] Wilson H Minimal physiological conditions for binocular rivalry and rivalry memory Vis Res 2007 3 2741 2750 10.1016/j.visres.2007.07.007 17764714

[B29] Blasdel GG Orientation selectivity, preference, and continuity in monkey striate cortex J Neurosci 1992 3 3139 3161 1322982 10.1523/JNEUROSCI.12-08-03139.1992 PMC6575662

[B30] Golubitsky M Shiau L-J Torok A Bifurcation on the visual cortex with weakly anisotropic lateral coupling SIAM J Appl Dyn Syst 2003 3 97 143 10.1137/S1111111102409882

[B31] Dias APS Rodrigues A Hopf bifurcation with $S_{N}$-symmetry Nonlinearity 2009 3 627 666 10.1088/0951-7715/22/3/007

[B32] Wilson H Computational evidence for a rivalry hierarchy in vision Proc Natl Acad Sci USA 2003 3 14499 14503 10.1073/pnas.2333622100 14612564 PMC283620

[B33] Wilson H Blake R Lee S Dynamics of traveling waves in visual perception Nature 2001 3 907 910 10.1038/35091066 11528478

[B34] Diekman C Golubitsky M McMillen T Wang Y Reduction and dynamics of a generalized rivalry network with two learned patterns SIAM J Appl Dyn Syst 2012 3 1270 1309 10.1137/110858392

[B35] Blake R Yu K Lokey M Norman H What is suppressed during binocular rivalry? Perception 1980 3 223 231 10.1068/p090223 7375329

